# Treatments for early-stage Dupuytren’s disease: an evidence-based approach

**DOI:** 10.1177/17531934221131373

**Published:** 2023-01-13

**Authors:** Jagdeep Nanchahal, James K.-K. Chan

**Affiliations:** 1 Kennedy Institute of Rheumatology, Nuffield Department of Orthopaedics, Rheumatology and Musculoskeletal Sciences, University of Oxford, Oxford, UK; 2 Department of Plastic Reconstructive Surgery, Stoke Mandeville Hospital, Buckinghamshire Healthcare NHS Trust, Aylesbury, UK

**Keywords:** Dupuytren’s disease, early-stage, evidence-based, anti-TNF, steroids, radiotherapy

## Abstract

Current treatments for Dupuytren’s disease are limited to late-stage disease when patients have developed flexion contractures and have impaired hand function. They all have limitations, including the risk of recurrence and complications. The use of treatments for early-stage disease, such as intralesional steroid injections or radiotherapy which lack a clear biological basis or evidence of effectiveness based robust randomized, double blind, placebo-controlled trials, highlights the desire of patients to access treatments before they develop significant flexion contractures. A detailed understanding of the cellular landscape and molecular signalling in nodules of early-stage disease would permit the identification of potential therapeutic targets. This approach led to the identification of tumour necrosis factor (TNF) as a target. A phase 2a clinical trial identified 40 mg in 0.4 mL adalimumab as the most efficacious dose and a subsequent randomized, double blind, placebo-controlled phase 2b trial showed that four intranodular injections at 3-month intervals resulted in decrease in nodule hardness and size on ultrasound scan at 12 months, and both parameters continued to decrease further at 18 months, 9 months after the final injection. This type of approach provides clinicians with a robust evidence base for advising their patients.

## The early-stage Dupuytren’s disease problem

A 60-year-old man presents with early-stage Dupuytren’s disease affecting his right dominant ring finger. He has a nodule just distal to the distal palmar crease and another nodule proximal to the proximal interphalangeal (PIP) joint crease. The nodules have been present for 8 years but in the last 6 months the nodule affecting the PIP joint has enlarged and become itchy and tender. He is a keen squash player and finds that the discomfort associated with the distal nodule impairs his racquet grip. On closer questioning he discloses that his father also had Dupuytren’s disease, developed recurrence following fasciectomy and subsequently underwent dermofasciectomy. The fragility of the full-thickness skin graft and cold sensitivity meant that his father had to stop working as a bricklayer.

Examination of the hand reveals full active extension of the metacarpophalangeal (MCP) joint and a 10° active extension deficit of the PIP joint that can be passively corrected to 5°. The nodules measure approximately 0.7 cm in diameter and there is a cord associated with the digital nodule, extending distally in the mid-axis towards the PIP joint and also proximally towards the base of the finger. There is no palpable cord associated with the proximal palmar nodule. The patient’s general practitioner has advised that he should wait for the finger flexion deformity to progress until it impairs hand function before considering surgery. However, the patient is keen to explore any available options to treat his early-stage Dupuytren’s disease and prevent progression.

## Introduction

The prevalence of Dupuytren’s disease had been reported to vary widely according to geographical location, with higher rates in populations from northern Europe (Hindocha et al., 2009). A systematic review and meta-analysis of populations in a range of Western countries found that risk is associated with age, 12% of those aged 55 years, 21% of individuals aged 65 years and 29% of people aged 75 years being affected (Lanting et al., 2014). Applying these figures to the corresponding age groups in the UK, we estimated that more than 5 million individuals over the age of 50 years would be affected (Dakin et al., 2022), which translates to approximately a prevalence of about 8% in the UK population. A recent study from the Netherlands found that 81% of patients with Dupuytren’s disease had ‘early-stage’ disease (Lanting et al., 2013). Applying this figure to the UK population would mean that approximately 4 million people have early-stage Dupuytren’s disease (Dakin et al., 2022). Not all individuals diagnosed with early-stage disease will develop flexion deformities and impairment of hand function. Van den Berge et al. (2021) found that flexion deformities developed in 20% of affected individuals over 7 years and Gudmundsson et al. (2001) reported that they occurred in about 35% at 18 years . We do not currently have any reliable method of predicting progression of disease, although those with Dupuytren’s diathesis (Hueston, 1993) (positive family history, ectopic disease, bilateral disease) are more likely to progress (Geoghegan et al., 2021). Based our recent trial (Nanchahal et al., 2022), which only included participants with progressive early-stage disease, we estimate that 2.5 million people would fall in this category, namely approximately 4% of the population. These figures illustrate the scale of the unmet medical need.

Current guidance is that intervention for Dupuytren’s disease should be delayed until there is fixed flexion deformity at the MCP joint of 30° or 15° at the PIP joint (Boe et al., 2021), with impairment of hand function. Consequently, patients presenting with earlier-stage disease are advised to wait until the disease progresses. Dupuytren’s disease often runs in families, with an estimated heritability of 80% (Larsen et al., 2015), and the prevalence across different ethnic populations is largely accounted for by 26 risk variants (Riesmeijer et al., 2019). Many patients with early-stage disease are, therefore, aware of the long-term outcomes through the experience of family members and would prefer to undergo treatment that delays or prevents progression rather than wait until the contracture of late-stage disease is established.

## Treatments for late-stage Dupuytren’s disease

The commonest procedure for late-stage Dupuytren’s disease remains excision of the diseased tissue (fasciectomy), although less invasive interventions, such as needle fasciotomy, have become increasingly popular due to the more rapid return of function (Bainbridge et al., 2012). Collagenase *Clostridium histolyticum* (CCH) also permits more rapid recovery and has become increasingly popular in the USA (Lipman et al., 2017), although it is now not readily available outside the USA.

There is currently a lack of high-quality comparative data of outcomes of the various treatment options (Rodrigues et al., 2015). The disease recurs in 21% of patients after surgical excision (fasciectomy) and in 85% after needle fasciotomy at 5 years (van Rijssen et al., 2012). Treatments for late-stage disease are also associated with potential complications, ranging from transient swelling and bruising through to nerve and tendon injury (Krefter et al., 2017). The surgical procedures of fasciectomy and dermofasciectomy are associated with overall local complication rates of about 12% and 17%, respectively, and include the more severe complications of nerve injury (3–6%), joint stiffness (10%) and complex regional pain syndrome (CRPS) (4–10%). The less invasive procedures of needle fasciotomy and CCH have higher overall complication rates of 19% and 78%, respectively, but most of these are relatively minor and transient. The risk of serious systemic complications, such as lower respiratory tract or urinary infections, pulmonary embolism and all-cause mortality, is low at 0.78% (Alser et al., 2020). Taken together, these data emphasize the need for effective, safe treatments for early-stage Dupuytren’s disease that would avoid the need for subsequent procedures.

## Treatments for early-stage Dupuytren’s disease

A systematic review of non-surgical treatments for early-stage Dupuytren’s disease identified various pharmacological treatments (oral, intramuscular, topical or intralesional steroids; vitamin E, furazolidone, aminosyn (an amino acid solution), hyperbaric oxygen), radiotherapy and physical therapies (ultrasound, splinting, frictional massage or heat treatment with joint stretching) (Ball et al., 2016). While all the studies were observational with a high risk of bias and were graded as Level 4 or 5 according to the Oxford Centre for Evidence Based Medicine grading, the best available data were for intralesional steroid injections or radiotherapy.

### Steroids

The rationale for intranodular and intralesional steroid injections was based on early clinical and experimental studies examining the inhibitory effect on connective tissue development (Zachariae and Zachariae, 1955), and subsequently on degradation of mature collagen in hypertrophic scars (Ketchum and Donahue, 2000). The earlier studies each reported less than 10 cases, with no blinding and lacked objective assessment of outcomes. A retrospective review of 63 patients (75 hands) with early Dupuytren’s disease is the largest available to date (Ketchum and Donahue, 2000). Patients received an average of 3.2 intranodular injections of 80–120 mg triamcinolone acetonide at each site at 6-weekly intervals. After 6 months, three further injections were administered if required. Follow-up ranged from 30 months to 27 years. Nodules were described as ‘easier to inject’ and to show 60–80% regression as defined by the nodules being ‘flatter’ in 73 hands, with no change in digital contracture. Disease reactivation requiring one or more further injections occurred in 50% of patients 1–3 years after the last injection. Adverse events, including transient depigmentation or subcutaneous atrophy at the injection site, were reported in 50% of patients, although all were reported as having resolved within 6 months of the last injection. A prospective study in Chinese patients from Taiwan reported the results of a single dose of 5 mg triamcinolone acetonide injected into nodules monthly for 3 months (Yin et al., 2017). Thirty-seven patients (49 affected hands) with early-stage Dupuytren’s disease were included, with an average follow-up of 5 years. The authors reported no progression of nodule size 6 months after injection on ultrasound scan. Reactivation of the treated nodules occurred in two patients (3 hands – 6%) after an average of 5 years, although none required surgical intervention. Nodules were reported to reduce in significantly size by 40% 6 months after injection and by 56% at the final follow-up. All the studies reporting the effect of intralesional steroids were confounded by the lack of a control group and lack of blinding, leading to potential assessor bias.

### Radiotherapy

Radiotherapy is usually directed against cells that are cycling. While the precise mechanism of action remains unclear, it is believed that radiotherapy reduces the development and growth rate of fibroblasts (Nice, 2016) and myofibroblasts (Finney, 1955; Keilholz et al., 1996). However, there is limited evidence of clinical effectiveness to date. Our systematic review (Ball et al., 2016) identified ten studies that investigated the primary treatment of radiotherapy in early-stage disease and all were limited by lack of quality, with no blinding, randomization or a control group and by the use of subjective outcome measures. Participant numbers were small (ten or fewer) in four (Corsi, 1966; Fenney, 1953; Finney, 1955; Grenfell and Borg, 2014). In the remaining six studies, two studies reported improvement (Keilholz et al., 1996; Lukacs et al., 1978), three were equivocal (Adamietz et al., 2001; Hesselkamp et al., 1981; Weinzierl et al., 1993) and one showed no change (Kohler, 1984). One group noted that their outcomes did not differ clearly from the natural history of early Dupuytren’s disease (Weinzierl et al., 1993). The National Institute for Health and Care Excellence (NICE) report considered safety, including acute and chronic toxicity (NICE, 2016). Assessment of acute toxicity by the NICE report included a randomized controlled trial (RCT) of 129 patients (198 hands) that compared two radiotherapy regimens; dry skin or redness (38%), dry desquamation (5%), wet desquamation (2%) and extensive erythema (6%), pronounced swelling (2%) were reported at 4 weeks follow-up. The same RCT reported chronic toxicity in 5% of hands within 12 months. A case series of 135 patients followed for a median of 13 years reported dry skin and desquamation in 23% and mild skin atrophy with occasional telangectasia in 7%. It was noted that there were no reports of radiation-induced malignancy. An ongoing trial seeks to evaluate the effectiveness of radiotherapy in patients with early-stage Dupuytren’s disease as well as for prevention of recurrence after treatment for late stage disease (DEPART, 2018). For the early-stage component, the investigators will recruit 372 participants aged >30 years with flexion contracture of <10°, evidence of progression of disease and no previous treatment for Dupuytren’s disease. The primary outcome will be based on progression of disease defined as the number of participants who develop flexion deformities of >20° or require intervention (surgery of needle aponeurotomy) assessed at intervals (6, 12, 24, 36, 48 and 60 months) over a 5-year period. Secondary outcomes will comprise patient-reported pain on a visual analogue scale, patient-reported outcome measures (Quick Disabilities of the Arm, Shoulder and Hand (Quick DASH) and Unité Rhumatologique des Affections de la Main (URAM)) and, where available, surface mapping of the disease. Participants will be randomized to observation or 30 Gray radiotherapy over ten fractions. The DEPART trial will also recruit participants who have been treated for late-stage disease (fasciectomy (*n* = 372), collagenase (*n* = 208) or needle aponeurotomy (*n* = 168)) to investigate the effectiveness of radiotherapy to control disease recurrence. While the trial will contribute much needed data to the field, disappointingly, there is no blinding and hence a risk of bias, especially with regards to patient-reported outcome measures.

### Collagenase

A randomized, double-blind placebo controlled trial of collagenase (Costas et al., 2017) for early-stage Dupuytren’s disease was not included in the systematic review of early-stage treatments (Ball et al., 2016), as collagenase is not approved for early-stage disease. The authors of the trial randomized 76 participants to a single injection of placebo (*n* = 17), collagenase 0.25 mg (*n* = 23), 0.4 mg (*n* = 18) or 0.6 mg (*n* = 18). Participants were assessed at 4 and 8 weeks. The surface area of the nodules assessed using callipers was significantly lower in the 0.6 mg and 0.4 mg collagenase groups compared with the placebo group, but not the 0.25 mg group. Nodule hardness assessed using a durometer was also reduced in all the collagenase groups. Assessment of nodule size using ultrasound scan proved unreliable and the authors recommended pre-specified rules for the use of this modality for future studies.

### Extracorporeal shock wave therapy (ESWT)

A recent publication reported the results of a blinded randomized trial of ESWT (*n* = 27) with placebo (*n* = 25) in participants with painful Dupuytren’s nodules followed for a total of 18 months (Knobloch et al., 2021). The rationale for the trial were previous reports of reduction in pain after ESWT in patients with Ledderhose or Peyronie’s disease. No evidence for the mechanism was provided and authors hypothesized that ESWT may affect TGF-β signalling, stem-cell propagation, growth factor stimulation or modulation of pain pathways via COX2, substance P or calcitonin gene-related peptide (CRGP). There was significant (*p < *0.05) reduction in pain in the ESWT group based on visual analogue scale, but no significant changes in patient-reported outcome measures (Michigan Hand Questionnaire, DASH or URAM).

## Anti-tumour necrosis factor: a targeted molecular approach from bench to bedside

All the treatments described above for the treatment of Dupuytren’s disease have been used in patients empirically, with no clear biological basis. The presumed mechanisms of action have been extrapolated from other studies, without any direct evidence for how they might translate to early-stage Dupuytren’s disease. A more rational approach would be based on assessment of the efficacy of treatments directed against targets that have been identified through a systematic understanding of the cellular and molecular basis of the pathogenesis of Dupuytren’s disease.

The development of all fibrotic diseases, including Dupuytren’s disease, is invariably preceded by inflammation (Wick et al., 2013). We defined the cellular composition of nodules of Dupuytren’s disease based on single cell profiling and identified populations of stromal and immune cells. The stromal cells comprised fibroblasts, myofibroblasts and vascular cells (Layton et al., 2020). Each group contained important subsets with different functions. The fibroblasts contained an ICAM1^+^ immune regulatory population characterized by high expression of the cytokine IL-6 and the chemokine CXCL8 (IL-8), and were found to promote chemotaxis of immune cells. The *ACTA2^+^* myofibroblasts were also not homogeneous and included cells along an activation continuum, ranging from fibroblast-like cells through to highly contractile CD82^+^ cells, together with a small but distinct cycling population. The immune cells included macrophages, mast cells, T cells and small numbers of B cells and neutrophils expressed receptors for a variety of chemokines, including CXCL8 (Izadi et al., 2019). Freshly disaggregated primary cells from Dupuytren’s nodules, which include all the representative cell populations, secreted several chemokines into the supernatant, including high levels of CXCL8 (Izadi et al., 2019). Freshly disaggregated nodular cells also secreted a variety of cytokines, including IL-6, TGF-β1, TNF and IL-1β (Verjee et al., 2013). Predictably, high, non-physiological, levels (1–10 ng/mL) of exogenous TGF-β1 converted all fibroblasts, irrespective of their origin, into highly contractile myofibroblasts. By contrast, TNF, at the levels secreted by freshly disaggregated nodular cells (∼50 pg/mL), selectively converted fibroblasts from the palm of patients with Dupuytren’s disease into myofibroblasts. Palmar fibroblasts from control, namely non-Dupuytren’s individuals and non-palmar fibroblasts from Dupuytren’s patients were unaffected at these concentrations; higher concentrations of TNF inhibited contractility of these cells. The importance of Wnt signalling pathways in Dupuytren’s disease has been identified in multiple Genome Wide Association Studies (GWAS) (Dolmans et al., 2011; Ng et al., 2017) and we found that TNF signalled via the canonical Wnt pathway to promote the development of the myofibroblast phenotype only in palmar fibroblasts from individuals with Dupuytren’s disease (Verjee et al., 2013). The selective effects on palmar fibroblasts from Dupuytren’s patients is an important observation as the disease predominantly affects the palm of genetically susceptible individuals and we subsequently showed the importance of epigenetic signalling in Dupuytren’s disease (Williams et al., 2020). Unlike TNF, other proinflammatory cytokines IL-6 and IL-1β had no effect on the contractility of any of the cells. Anti-TNF led to dose-dependent inhibition of the myofibroblast phenotype and, of the clinically approved agents, the fully human IgG molecules adalimumab and golimumab were the most efficacious in vitro at the doses tested (Verjee et al., 2013). More recently, we have shown that endothelial cells maintain the ICAM1^+^ immune regulatory fibroblasts via platelet derived growth factor (PDGF) signalling in a perivascular niche (Layton et al., 2022). Based on these findings, we propose a model for Dupuytren’s disease nodules in which these ICAM1^+^ fibroblasts secrete chemokines, which attract immune cells, including M2 macrophages and mast cells (Izadi et al., 2019). These in turn secrete low levels of TNF that promotes the development of myofibroblasts (Verjee et al., 2013). The latter secrete low levels of IL-33, which acts on the immune cells to maintain the chronic expression of TNF (Izadi et al., 2019). Myofibroblasts coordinate their activities via intercellular junctions to effectively act as a syncytium (Verhoekx et al., 2013). This detailed understanding of the complete cellular ecosystem of Dupuytren’s nodules illustrates the division of labour of the different cell populations and how they interact via a complex network of local signalling pathways and cross-talk to maintain the chronic low-grade inflammation that is responsible for the development of Dupuytren’s disease in genetically susceptible individuals.

Having identified TNF as a potential therapeutic target, we postulated that intranodular injections of adalimumab may be efficacious in controlling disease progression (Nanchahal et al., 2017). This would be consistent with our understanding of Dupuytren’s disease as a low-grade localized chronic inflammatory disorder, without a concomitant increase in systemic levels of circulating cytokines (Izadi et al., 2019). The optimal dose and concentration of adalimumab for intranodular injection was defined in a phase 2a clinical trial based on an experimental medicine design (Nanchahal et al., 2017). Patients with prominent Dupuytren’s nodules who were scheduled to undergo surgery 2 weeks later were recruited and the nodules were injected with varying doses of adalimumab or an equivalent volume of saline. The excised nodules were then analysed for markers of myofibroblast activity. Only 40 mg of adalimumab in 0.4 mL was found to lead to significant downregulation of α-smooth muscle actin and procollagen type I proteins; 15 mg of adalimumab in 0.3 mL and 35 mg in 0.7 mL were not efficacious (Nanchahal et al., 2018). We noted that some of the material extravasated out of the nodule into the subcutaneous tissues when we injected a volume of 0.7 mL, suggesting that high local doses would be the most efficacious. Next, we proceeded to a double blind, placebo controlled randomized phase 2b clinical trial (Nanchahal et al., 2022), We recruited 140 participants in the UK and 34 in the Netherlands with early-stage Dupuytren’s disease, a well-defined nodule and a clear history of disease progression. Participants were randomized 1:1 to receive either four intranodular injections of 40 mg adalimumab in 0.4 mL or an equivalent volume of saline every 3 months. The 3-month intervals were selected based on a patient survey of acceptability. The primary outcome was nodule hardness at 12 months compared with baseline, 3 months after the participants received their final, fourth injection. Participants were followed for 18 months from baseline. Secondary outcomes included nodule size on ultrasound scan, patient-reported outcome measures (PROMS) (Michigan Hand Questionnaire (MHQ) and most affected activity), extension deficit of the joint affected by the study nodule and grip strength. The study met the primary endpoint, with a statistically significant decrease in nodule hardness (*p = *0.0002). Nodule area on ultrasound scan was also decreased at 12 months (*p = *0.0025). Both nodule hardness (*p < *0.0001) and area (*p < *0.0001) continued to decrease further at the 18-month time point, 9 months after the final injection. Patients with early-stage disease have little impairment of hand function and, predictably, there was no change in PROMS of hand function. There was no statistically significant change in passive extension deficit of the affected MCP joints over the 18-month course of the trial, although passive extension deficit was better when nodules that affected PIP joints were treated with adalimumab. However, the number of PIP joints was small (at baseline adalimumab *n* = 16, saline *n* = 10). More participants in the saline group (*n* = 10) had undergone or were awaiting surgery than the adalimumab group (*n* = 3); again, the overall numbers were small and precluded statistical analysis. Based on previous studies that estimated that 20% of patients with Dupuytren’s disease experience progression to develop finger contractures over 7 years and 35% over 18 years (Gudmundsson et al., 2001; van den Berge et al., 2021), follow-up for approximately 10 years would be required to ascertain whether intranodular injection of adalimumab and the observed statistically significant reduction in nodule hardness and nodule size on ultrasound scan would impact on the development of finger deformities and impact hand function as assessed by PROMs, such as MHQ. There were no related severe adverse events and there was no difference between the saline and adalimumab groups with respect to minor local injection site reactions (local itching, redness, haematoma, bruising, blistering).

Taken together, the in vitro (Izadi et al., 2019; Verjee et al., 2013) and phase 2a trial data (Nanchahal et al., 2018) show that anti-TNF downregulates the phenotype of myofibroblasts from Dupuytren’s nodules. The phase 2b clinical trial data suggest that intranodular injections of adalimumab may be efficacious in delaying or preventing the progression of early-stage Dupuyten’s disease although follow-up over approximately 10 years would be required to confirm this (Nanchahal et al., 2022). Each active nodule would need to be injected and the series of four injections would need to be repeated if the nodule were to re-activate.

## Conclusions

Patients with early-stage Dupuytren’s disease are currently advised to await progression to late-stage disease before being offered treatments, such as needle fasciotomy, collagenase injections or surgery, all of which have limitations. There is a pressing need for safe, effective treatments for early-stage disease. There are numerous reports of a variety of treatments that have been used empirically, with no clear biological basis for their use in Dupuytren’s disease. Clinical outcomes are largely reported with no control groups or blinding, making it impossible for clinicians to advise patients on the relative merits of each treatment. In recent years, a detailed understanding of the molecular basis of disease has transformed medical practice. An example is rheumatoid arthritis, where 20 years ago patients would routinely undergo synovectomy in an attempt to control disease, which invariably recurred. The identification of TNF as a therapeutic target (Brennan et al., 1989) transformed rheumatological practice (Feldmann and Maini, 2003) and dramatically reduced the necessity for surgical intervention. In 1833 Dupuytren noted ‘the multitude of reasons assigned as a cause, the quantity of remedies proposed for its cure … . Authors have spoken in a very incomplete manner upon this subject’ (Dupuytren, 1834). As hand surgeons we are well-placed to access diseased and control tissues and apply advanced molecular biological techniques to unravel the key signalling mechanisms responsible for disease pathogenesis. The identification of TNF, a therapeutic target and the of efficacy of treatment in double blind randomised controlled trials, is an example of using this rational approach to allow us to advise and treat patients in a truly evidence-based manner.
